# Metastasis is an early event in mouse mammary carcinomas and is associated with cells bearing stem cell markers

**DOI:** 10.1186/bcr3102

**Published:** 2012-01-25

**Authors:** Desheng Weng, Jeffrey H Penzner, Baizheng Song, Shigeo Koido, Stuart K Calderwood, Jianlin Gong

**Affiliations:** 1 Department of Medicine, Boston University School of Medicine, 650 Albany Street, Boston, MA 02118, USA; 2 Department of Internal Medicine, Jikei University School of Medicine, 163-1 Kashiwa-shita, Kashiwa, Chiba 277-8567, Japan; 3 Molecular and Cellular Radiation Oncology, Beth Israel Deaconess Medical Center, Harvard Medical School, 99 Brookline Avenue, Boston, MA 02215, USA

## Abstract

**Introduction:**

It is still uncertain whether metastasis is predominantly an early or late event in tumor progression. The detection of early metastases and cells responsible for the dissemination may therefore have significant clinical implications.

**Methods:**

Lung dissemination and/or metastasis were investigated in mice carrying the polyomavirus middle-T oncogene (PyMT) during different stages of mammary tumorigenesis using the colony forming assay. Immunocytochemical or immunohistochemical staining was used to identify subpopulations of cells responsible for lung dissemination and metastasis. Histological examination was used to show primary and metastatic tumors. The tumor-initiating and metastatic capacity of cells expressing stem cell markers was assessed in syngeneic wild-type (WT) mice whose mammary fat pads were injected with these cells.

**Results:**

Metastatic mammary epithelial cells were detected in the lungs of mice carrying the PyMT oncogene (MMT mice). These cells were observed early in breast tumorigenesis when the mammary tree appeared by histological inspection to be normal (or at a premalignant stage), suggesting the possession of disseminating and metastatic capacity even before full malignant transformation. Some of the disseminated cells and lung metastases displayed surface stem cell markers. These findings suggest that stem cells from apparently precancerous primary lesions could be a source of metastasis. Indeed, injection of lung tissue cells from MMT mice into syngeneic WT mice resulted in the formation of mammary tumors. These tumors resembled their parent mammary tumors in the MMT donors as well as grafted tumors derived from mammary tumor cells. Furthermore, when we injected lung tissue cells from GFP MMT mice into the fat pads of recipient WT mice, disseminated or metastatic GFP-expressing cells were detected in the lungs, lymph nodes and blood of the recipient WT mice. We finally identified a subpopulation of mammary epithelial/tumor cells expressing CD44 and Sca1 that was largely responsible for dissemination and metastasis in MMT mice.

**Conclusions:**

The tumorigenic and metastatic potential of a subpopulation of mammary epithelial/tumor cells in MMT mice is endowed relatively early in mammary neoplasms and suggests a potential role for cancer stem cell sub-populations in metastasis.

## Introduction

Metastasis is a complex, multi-step process in which cells from primary tumors invade surrounding tissues, intravasate into the circulation (circulating tumor cells), arrest in the capillary beds, and extravasate from the circulation into the distant organ. These disseminated tumor cells may remain in a quiescent state in the new home but then proliferate and develop into vascularized metastatic tumors years later in a process stimulated by unknown factors that may include signals received from the environmental niche [1,2]. Some prevailing models of metastasis contend that genetic mutations accumulating late during multi-stage tumorigenesis provide a selective advantage that permits tumor cells to metastasize [3]. In support of this notion is the clinical observation that metastasis is often diagnosed in primary tumors with a diameter of more than 2 cm and early surgery often cures the disease [4,5]. Thus, metastatic capacity is considered a relatively late process in tumor progression [6]. However, contrasting models of metastasis propose that some tumor cells may possess metastatic properties in the earlier stages of tumorigenesis [7,8]. Metastasis appears to be an inefficient process. For example, although 90% of intravenously injected melanoma cells could colonize the liver, only about 0.02% of the cells developed into macrometastases [9]. Thus, the metastatic potential of tumor cells entering the circulatory system is not necessarily uniform and only a few cells possess the capacity to migrate to the remote organs, proliferate, and become metastatic tumors. Although the origin and true identity of the metastatic cells capable of forming macrometastasis remain elusive, recent studies indicate that subpopulations of tumor cells with tumor-initiating ability are candidates because they can survive in foreign microenvironments and evolve into heterogeneously metastatic tumor [8]. These cells are sometimes called cancer stem cells (CSC) because they also express markers detected on the surfaces of normal tissue stem cells. CSC are sub-populations of tumors found both in hematopoietic malignancies [10,11] and solid tumors, including breast cancer [12-15]. CSC possess the properties of self-renewal and multipotency that may be critical for the survival of disseminated cells and development of heterogeneous macrometastasis. In addition, recent studies provide evidence that cells from premalignant lesions and ductal carcinomas *in situ *(DCIS) can migrate and develop into fully developed malignant tumors [16,17]. Precancerous stem cells (pCSC) have been identified in preleukemic clones, mammary intraepithelial neoplasia outgrowths (premalignant lesions) and precancerous stem cell lines [18-20]. Injection of pCSC into SCID mice resulted in the development of benign or malignant tumors [18]. Despite these findings, it remains uncertain at which stage a developing cancer or precancerous cell becomes endowed with metastatic capacity. In addition, the cell subpopulations responsible for early metastasis have not been characterized. It is, however, evident that the identification of cells responsible for initiating metastasis has significant clinical implications. Targeted therapy against these cells would become feasible.

We previously investigated the role of telomerase and telomere maintenance in mammary tumorigenesis and metastasis in mice that carry the polyomavirus middle T (PyMT) oncogene (MMT mice). Metastasis was found in lung tissue collected from telomerase-proficient MMT mice, whereas we failed to detect tumor metastasis in the sections of lungs from telomerase-deficient MMT mice [21], suggesting that distant metastasis in MMT mice requires telomerase activity. We found in these studies that MMT mice are reliable tumor models with predictable rates of mammary tumor growth and lung metastasis. In the present study, we have investigated metastasis triggering events that may occur early in tumorigenesis and attempted to identify cell sub-populations potentially responsible for metastasis. Our experiments show that subpopulations of premalignant mammary epithelial cells (MECs) developing in these mice are endowed with the capacity of dissemination. In addition, their ability to disseminate and form tumors could be maintained after transfer of the primary malignant cells to a new host devoid of overt oncogene expression. Furthermore, a subset of cells bearing CSC markers in the primary and metastatic lesions could be detected during the various stages of mammary tumorigenesis and they appeared to play an important role in tumor dissemination and metastasis. The present studies therefore support a model of parallel development of primary and secondary tumors.

## Materials and methods

### Mice

The mice (C57BL/6 background) used in our experiments include female mice transgenic for the PyMT (MT mice) oncogene driven by the mouse mammary tumor virus long terminal repeat (MMTV-LTR) and MMT mice double transgenic for PyMT and the human MUC1 antigen (mucin 1) [22,23] (a kind gift from Sandra J. Gendler, Mayo Clinic, Scottsdale, AZ, USA). Mice expressing PyMT develop mammary carcinomas [24], and the MUC1 antigen is expressed in a tissue-specific fashion similar to that in humans [22]. GFP mice (C57BL/6-Tg, CAG-EGFP) were purchased from the Jackson Laboratory (Bar Harbor, MN, USA) and crossed over MMT mice to generate GFP MMT mice. Wild-type (WT) female C57BL/6 mice (C57BL/6NTac) were purchased from Taconic Farms (Germantown, NY, USA) and used as recipient mice to determine the tumorigenic and metastatic potential of cells isolated from lungs or mammary glands of MMT mice. Animals were maintained in microisolator cages under specific pathogen-free conditions. The use of mice was approved by the Institutional Animal Care and Use Committee of Boston University Medical Center.

### PCR

PCR analysis was used to confirm the presence of the MUC1, PyMT and GFP genes in transgenic mice. Tail tissue DNA was extracted using the REDExtrac-N-Amp Tissue PCR Kit (Sigma, Steinheim, Germany). A 100 nM sample of 5'-AGTCACTGCTACTGCACCCAG-3' forward primer and 5'-CTCTCCTCAGTTCCTCGCTCC-3' reverse primers were used for the MT gene and 5'-CTTGCCAGCCATAGCACCAAG-3' and 5'-CTCCACGTCGTGGACATTGATG-3' for the MUC1 gene. Primers for the detection of GFP gene include 5'-AAGTTCATCTGCACCACCG-3' (forward), 5'-TCCTTGAAGAAGATGGTGCG-3' (reverse), and internal positive control 5'-CTAGGCCACAGAATTGAAAGATCT-3' (forward), 5'-GTAGGTGGAAATTCTAGCATC ATCC-3' (reverse). PCR was carried out using these primers as well as the additional reagents: 10 μl 2 × PCR mix, 4 μl tail DNA, and reagent quality water. Size fractionation in a 1.5% agarose gel was used to analyze the PCR products [23].

### Whole mount and H&E staining

Mice were sacrificed at the indicated ages. For whole-mount preparations, thoracic mammary glands (three pairs) were harvested and the resected tissue was spread onto a slide and fixed in Carnoy's fixative (60% ethanol, 30% chloroform, 10% glacial acetic acid) for two to four hours at 4°C. The tissue mount was then washed in 70% ethanol for 15 minutes, 50% ethanol for 15 minutes, rinsed with distilled water for five minutes and placed in a Carmine Alum staining solution over night. Stained whole mammary glands were kept in 70% ethanol at 4°C for photograph. The solid masses as indicated by the deep red-staining with Carmine Alum and greater than 1 mm^2 ^areas were measured using Spot Advanced™ digital imaging software (Diagnostic Instruments, Inc., Sterling Heights, MI, USA). For H&E staining, mammary glands were paraffin-embedded, sectioned (5 μm), stained with H&E, and examined under light microscope.

### Colony-forming assay for proliferating cells in lung tissue

To identify the presence of disseminated cells in the lungs, a 24 G needle was used to perfuse the lungs of blood with sterile PBS via the right ventricle of the heart before harvesting the lung tissue. The lungs were collected, minced and digested in a collagenase enzyme cocktail solution as previously described [21,25]. Single cells were cultured in 10% FCS Dulbecco's modification of Eagle's medium™ (DMEM) for two weeks and stained with anti-CD44 (clone IM7), anti-Sca1 (clone D7) (e-Bioscience, Inc., San Diego, CA, USA), anti-ER (clone MC-20), (Santa Cruz Biotechnology Inc., Santa Cruz, CA, USA) or anti-MUC1 (clone HMPV) (BD Pharmagen, San Diego, CA, USA) antibodies using standard immunocytochemical (ICC) staining. Presence of disseminated or metastatic tumor cells was determined by growth of tumor colonies on tissue culture plates and colonies were detected with 0.5% crystal violet staining. Each colony (> 50 cells) showing either MUC1 or GFP positivity was counted to quantify the number of disseminated cells for each individual mouse.

### Immunocytochemical staining

The mammary tumors and perfused lungs were minced and incubated overnight in DMEM with 10% FCS, 2 mM L-glutamine, and 100 μg/ml of both penicillin and streptomycin (Cellgro, Mediatech, Inc., Manassas, VA, USA) in a Heracell CO_2 _incubator at 37°C and 5% CO_2_. Lung tissue cells were purified using 5 ml Ficoll-Paque PLUS™ solution (GE Healthcare, Piscataway, NJ, USA) to remove dead cells and then stained with antibodies against CD44, Sca1, MUC1 and/or epithelial specific antigen (ESA, clone G8.8) (e-Bioscience, San Diego, CA, USA) using standard ICC staining method. A similar method was used to analyze the GFP-positive metastatic cells that co-expressed ESA in the blood, lymph nodes (LNs), and lungs of WT-recipient mice.

### Injection of lung tissue cells or mammary tumor cells into wild-type recipients

WT mice were anesthetized via intraperitoneal injection of ketamine (100 mg/kg) plus xylazine (10 mg/kg). Matrigel suspensions 1:1 of lung cells ranging in number from 1 × 10^3 ^to 1 × 10^6^, from MMT or GFP MMT mice at different stages of tumorigenesis were injected into mammary fat pads [26,27]. In some experiments, the CD44 and Sca1 double-positive (CD44/Sca1^+^) and double-negative (CD44/Sca1^-^) cells obtained by cell sorting from MECs, mammary tumors, or lungs of GFP MMT mice were transplanted to WT mice using the same method. The MECs were isolated according to the method previously described [28] with modifications. The mice were followed for up to two months, at biweekly intervals for the growth of tumors. At the end of experiment the mice were sacrificed and the lungs, draining LNs and mammary tumors, if any, were harvested and examined.

### Statistical analysis

For samples in which actual mean values could be attained, including mammary masses (mm^2^) and the number of lung cell colonies formed, one-way analysis of variance (ANOVA) was used to determine *P *values. *P *values of less than 0.05 were considered statistically significant. To compare percentages of positive-staining tumor cells between age groups of mice, the Chi-Square test was used to determine the *P *values. The statistical analysis software, SPSS Statistics™ v17.0 (IBM Corporation, Somer, NY, USA), was used to attain these values.

## Results

### Detection of disseminated cells or metastasis in the lungs of young MMT mice

**Figure 1:**
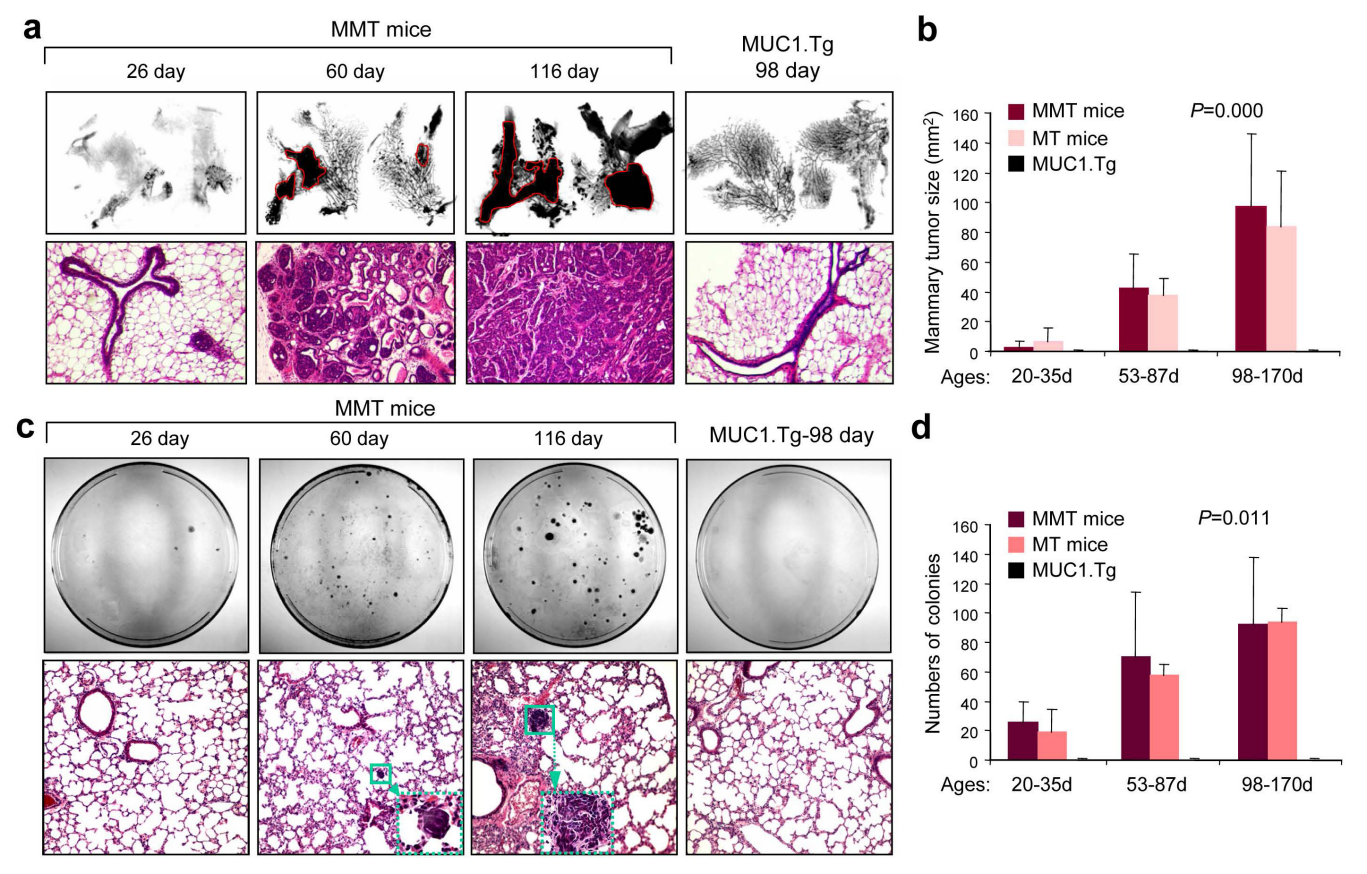
**Development of mammary tumors and detection of disseminated and/or metastatic cells in MMT mice**. (**a**) Mammary glands were harvested from mice double transgenic for PyMT and MUC1 antigen (MMT) or MUC1 transgenic (MUC1.Tg) mice at indicated ages, processed for whole mount or sections and stained with H&E. The solid masses are indicated by circles. (**b**) Comparison of mammary masses in different age groups (*n *= three to seven per group) of MMT, MUC1.Tg and MT (mice transgenic for PyMT oncogene) mice. The whole mount was digitized and the solid masses were traced and measured using SPOT advanced software. (**c**) Detection of disseminated cells or metastasis in lungs. Lungs were harvested from MMT mice at the indicated ages and perfused to remove circulating cells. The lung tissue was minced and digested in a collagenase enzyme cocktail solution. The cells were then cultured on tissue culture plates for two weeks and stained with 0.5% crystal violet (upper panels). Lungs from mice at the indicated ages were processed for histological examination with H&E staining. The green square indicates the enlarged area. (**d**) Colonies of more than 50 cells from lung cell cultures of each mouse (*n *= three to seven per group) were counted. Statistical analysis was performed using one-way analysis of variance.

MMT mice transgenic for PyMT and MUC1 develop mammary tumors in multiple stages (Figures 1a and 1b). The mammary trees in such mice appeared normal or hyperplastic at 26 days postnatal. Early hyperplastic lesions then evolved into defined solid masses at around an age of 60 days and finally became diffuse invasive tumors by 116 days (Figure 1a). H&E-stained sections from breast tissue at each of these ages are shown below and support these conclusions (Figure 1a, lower panels). By contrast, mammary trees in PyMT-negative MUC1 transgenic (MUC1.Tg) mice remain unchanged with few side branches and we did not observe overt tumorigenesis (Figure 1a; right panel). Based on our observations of mammary tumor development [21], MMT mice were arbitrarily stratified into three age groups for purpose of experiment, including: 20 to 35 days (hyperplastic lesion), 53 to 87 days (mammary intraepithelial neoplasms (MIN), a premalignant stage), and 98 to 170 days (malignant stage) and compared with control MT and MUC1.Tg mice. Significant increases in total solid tumor mass were observed in the breast tissues of the 53 to 87 days and 98 to 170 days groups (Figure 1b).

We next investigated the relative levels of viable disseminated cells using an *in vitro *colony-forming assay to detect cells with proliferative potential within the lung tissues. In general, the proportion of colony-forming cells in the lungs increased with the average age of these MMT mice (Figures 1c and 1d). Surprisingly, culture of lung tissue cells from mice as young as 26 days postnatal resulted in tumor colony formation (Figures 1c and 1d). Furthermore, metastatic tumors were found in mice at an age of 60 days (Figure 1c, lower panels). Before processing for colony-forming assay, lungs were carefully perfused to remove circulating cells [see Additional data file 1]. Thus, the colony-forming cells in the culture of lungs likely represent the disseminated and/or metastatic cells from the primary mammary epithelia/carcinomas as MMTV-PyMT expression has not been shown to lead to direct transformation of lung cells [24]. In repeated experiments, we failed to observe colony formation in lung tissue from the control MUC1.Tg mice, which do not develop breast carcinoma (Figures 1c and 1d). Consistent with previous findings [23], expression of MUC1 in MMT mice did not alter mammary tumorigenesis or metastasis since comparable tumor sizes and numbers of lung colonies were observed between MT and MMT mice (Figures 1b and 1d). These experiments suggest that lung dissemination or metastasis may be an early event in mammary tumorigenesis of MMT mice.

### Lung-derived cells from MMT mice contain a sub-population expressing CD44 and Sca1 with mammary tumor cell morphology

**Figure 2:**
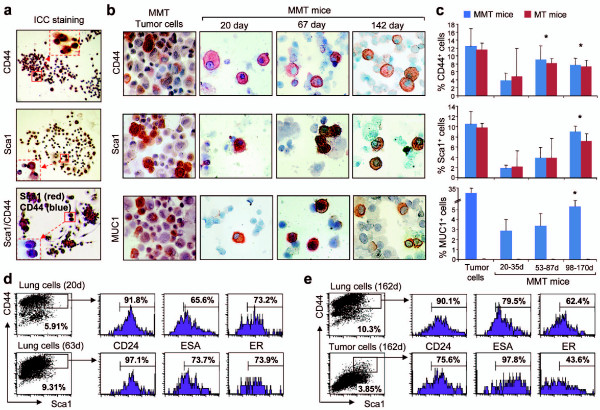
**Detection of cells expressing stem cell markers or MUC1 in the lungs of MMT mice**. (**a**) Immunocytochemical (ICC) staining. The colonies growing from lung tissue cells (as in Figure 1) were stained with anti-CD44 or Sca1 antibodies. The cells staining red were positive cells and those stained purple were considered double-positive cells (10x). The red square indicates the enlarged area. (**b**) Single-cell suspensions were made from the lungs of mice double transgenic for PyMT and MUC1 antigen (MMT) at the indicated ages and stained with monoclonal antibodies against CD44, Sca1 or MUC1 using ICC staining. Cells isolated from mammary tumors of MMT mice were used as positive control. Cells staining red were considered positive (60x). (**c**) Comparison of positive cells among different age groups (*n *= three to eight per group) of MMT and MT mice. The cells positive for the indicated antibody from each mouse were counted and are presented in the bar graph. Statistical significance between groups was determined using Chi-square test and the symbol "*" indicates *P *< 0.05 when 20 to 35 day group was compared with 53 to 87 day or 98 to 170 day groups. (**d and e**) Expression of CD24, ESA (epithelial specific antigen) or estrogen receptor (ER) on CD44/Sca1^+ ^cells. The lung cells isolated from MMT mice at the indicated ages were processed for staining with anti-CD44-Cy, Sca1-FITC and CD24-PE, ESA-PE or ER-PE antibodies. The percentage of cells double positive for CD44 and Sca1 were gated and then further analyzed by FACS for expression of CD24, ESA or ER. The percentage of gated CD44/Scal^+ ^cells in total lung or tumor cells and percentage of triple-positive cells are presented. (**e**) Mammary tumor cells isolated from 162-day-old MMT mice were used as controls.

We next further characterized the colony-forming cells in the lungs of MMT mice. Lung-derived colonies were stained with a panel of antibodies against cancer stem cell and mammary carcinoma markers. Surprisingly, most of the colony-forming cells were positive for the cancer stem cell markers CD44 and Sca1 (Figure 2a). In addition, the morphology of these colony-forming lung cells resembled that of primary mammary cancer cells in Figure 2b.

We next examined single cell suspensions prepared from the lungs of MMT mice at different stages of mammary cancer progression. Our hypothesis was, if CD44^+ ^and Sca1^+ ^cells are involved in metastasis to the lung from primary mammary carcinomas, we should be able to find such cells in the lungs. Single cell suspensions obtained from the lungs of MMT mice at increasing ages were therefore stained with monclonal antibodies against CD44, Sca1 or MUC1 and examined under microscopy. Primary mammary tumor cells from MMT mice were used as positive control in the left panels of Figure 2b. Red-stained CD44, Sca1, or MUC1-positive cells were identified among the lung tissue cells prepared from MMT mice at each age group, although an age-dependent increase in cells expressing each of these markers was observed (Figure 2c). Comparable numbers of CD44^+ ^or Sca1^+ ^cells were observed in each age group between MT and MMT mice, suggesting that the expression of MUC1 does not affect the pool of CD44^+ ^or Sca1^+ ^cells (Figure 2c). In addition, the majority of the CD44/Sca1^+ ^lung cells also expressed the surface markers CD24, ESA, or estrogen receptor (ER) at levels comparable with those expressed by primary tumor cells (Figures 2d and 2e). These experiments indicate that cells bearing stem cell, tumor or mammary epithelial markers can be identified in the lungs of MMT mice, suggesting that they originate from mammary epithelial/tumor cells and may be important seeding cells of lung metastasis.

### The tumorigenic potential of lung tissue cells from MMT mice

**Figure 3:**
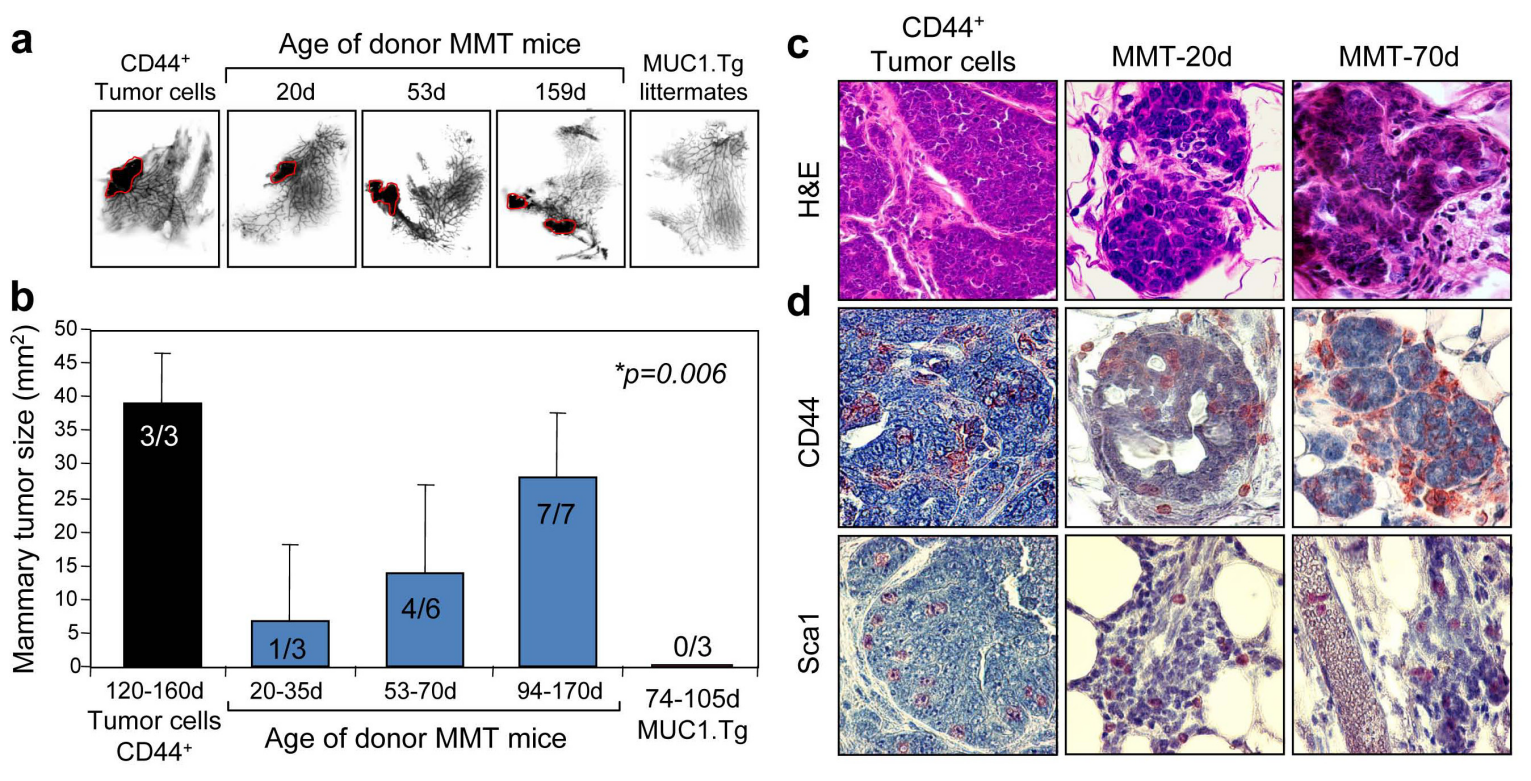
**Tumorigenic potential of lung tissue cells from MMT mice**. (**a**) Single-cell suspensions were isolated from lungs of mice double transgenic for PyMT and MUC1 antigen (MMT) at the indicated ages, counted and then injected into the left and right mammary fat pads of syngeneic wild-type (WT) mice at various doses. Eight weeks after inoculation, the recipient mice were sacrificed and the mammary glands were harvested, processed for whole mount and photographed. WT mice injected with sorted CD44^+ ^mammary tumor cells or single-cell suspension obtained from the lungs of MUC1.Tg mice were used as positive or negative controls, respectively. The solid masses indicated by circles are the tumors. (**b**) Tumor incidence and tumor size in the whole mount were summarized and compared using one-way analysis of variance. (**c and d**) The mammary glands as shown in (**a**) were processed for histology and (**c**) stained with H&E or anti-CD44 and Sca1 monoclonal antibodies by (**d**)immunohistochemical (IHC) staining.

We next assessed the tumorigenic potential of these lung tissue cells that possess the properties of colony formation and expression of CSC markers. Single cell suspensions obtained from the lungs of MMT mice at the various stages of mammary tumor progression were injected into the mammary fat pads of WT mice. A total of 22 WT mice were used, of which 16 received lung tissue cells from MMT mice, three WT mice received lung tissue cells collected from MUC1.Tg mice as negative controls, and three WT mice were injected with CD44^+ ^tumor cells sorted from primary MMT mammary tumors, as positive controls. As shown in Figures 3a and 3b, injection of lung tissue cells from MMT mice into the fat pads of WT mice resulted in tumor growth in 75% (12 out of 16) of the mice. By contrast, there was no evidence for mammary tumor formation in mice injected with lung cells from control MUC1.Tg mice. By stratifying MMT donor mice according to age, we were able to detect an age-dependent trend in tumor initiation potential in lung metastatic cells (Figure 3b). Of recipient mice, 33.3% developed mammary tumors when they were injected with lung tissue cells from MMT mice at ages between 20 and 35 days. In the other groups of WT mice, it was found that four out of six and all seven mice that received lung tissue cells from MMT mice at 53 to 70 days and 94 to 170 days old, respectively, developed mammary tumors (Figure 3b). Tumor formation in each group was further confirmed by histological examination (Figure 3c). By contrast, we detected no mammary tumor formation in WT mice injected with lung cells from MUC1.Tg control mice (Figure 3a). The morphology of tumors in WT mice derived from MMT mouse lung tissue cells resembled the primary mammary tumors in MMT mice (data not shown) as well as grafts of CD44^+ ^tumor cells into WT mice (Figure 3c). In addition, the level of CD44 and Sca1 expression in the grafted tumors was comparable with that in the grafts of CD44^+ ^tumor cells (Figure 3d). Interestingly, Sca1-positive cells could be identified inside blood vessels in tissue sections of the grafted tumor in WT mice injected with lung tissue cells from a 70-day-old MMT donor mouse (Figure 3d). Taken together, these results suggest that breast migrants and metastases in lung tissue contain a subpopulation of cells expressing stem cell markers with tumorigenic potential, that these cells migrate to lungs early in mammary tumorigenesis, and that lung metastasis increases with progression of the primary tumor.

### Inherited disseminating and metastatic potential in the transplanted lung tissue cells from MMT mice

**Figure 4:**
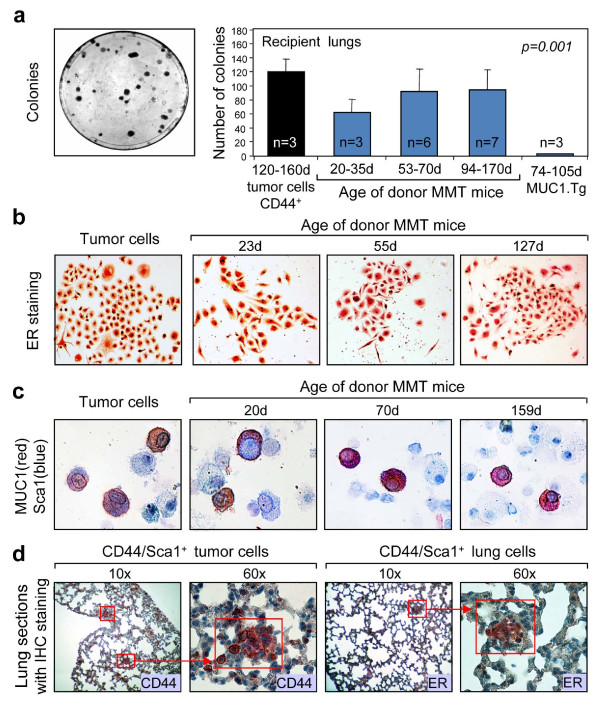
**Detection of disseminated and/or metastatic tumor cells in the new hosts**. Single-cell suspensions from the lungs of mice double transgenic for PyMT and MUC1 antigen (MMT) or MUC1 transgenic (MUC1.Tg) mice at indicated ages were injected into the fat pads of mammary glands of wild type (WT) mice. Mice injected with sorted CD44^+ ^mammary tumor cells were used as controls. Eight weeks after the inoculation, the lungs were harvested from the mice, perfused to remove the circulation cells, and then processed for clonogenic assay. (**a**) Colonies from lung cell cultures of each mouse were counted and are presented in the bar graph. Statistical significance among groups was compared using one-way analysis of variance. (**b**) Expression of estrogen receptor (ER) in some cells from colonies as shown in (**a**) using immunocytochemical (ICC) staining. (**c**) Detection of MUC1/Sca1 positive tumor cells in lung tissue cells isolated from WT mice using ICC staining. (**d**) Metastatic lesions in the lungs of WT mice injected with sorted CD44/Sca1^+ ^from mammary tumors (left two panels) or lungs of MMT mice (right two panels). The lungs of recipient mice were processed for immunohistochemical (IHC) staining with anti-CD44 or anti-ER monoclonal antibodies. The red square indicates the enlarged area.

We next determined whether disseminating and metastatic potential could be transferred from cells growing in the lungs of MMT mice to the mammary fat pads of WT mice. We then used the colony-forming assay to examine the levels of viable, proliferating cells in the lungs of these mice. Indeed lung colonies were observed after this procedure (Figure 4). Tumor colony frequency increased in the age of 53 to 70 day donor mice although a significant number of colonies were still observed in the lungs of WT mice receiving lung tissue cells from young MMT mice (Figure 4a). By contrast, we have observed minimal colony formation in the lung tissue cells of control WT mice injected with lung tissue cells from MUC1.Tg mice (Figure 4a). Some cells in the colonies were positive for ER expression (Figure 4b), suggesting a mammary epithelial origin. In addition, MUC1/Sca1 double-positive (red/blue) cells were also observed in the lung tissue cells of WT mice that were injected with mammary tumor cells or lung tissue cells from MMT donor mice at different age groups (Figure 4c). It should be noted that the MUC1 expression in these cells was diffuse and spread over the entire cell, one of the characteristics of mammary carcinoma cells in MMT mice (Figure 4c). To confirm whether CD44/Sca1^+ ^lung cells could seed metastasis, CD44/Scal^+ ^cells obtained by cell sorting from mammary tumors or lungs of MMT mice at ages of more than 120 days were injected into the WT mice. Lung metastasis was observed in mice injected with CD44/Scal^+ ^tumor cells (Figure 4d). In addition, the metastatic tumor expressed ER, confirming its mammary epithelial origin (Figure 4d, right panel). Together, these results indicate that lung tissue cells from MMT mice at various stages of tumor progression could result both in forming primary tumors in recipient fat pad and in seeding tumor dissemination and metastasis in recipient syngeneic WT mice. Our studies appear to suggest an inherent tumorigenic and metastatic capability within MMT cells bearing stem cell markers.

### Disseminating and metastatic potential of lung and mammary tumor cells from GFP MMT mice

**Figure 5:**
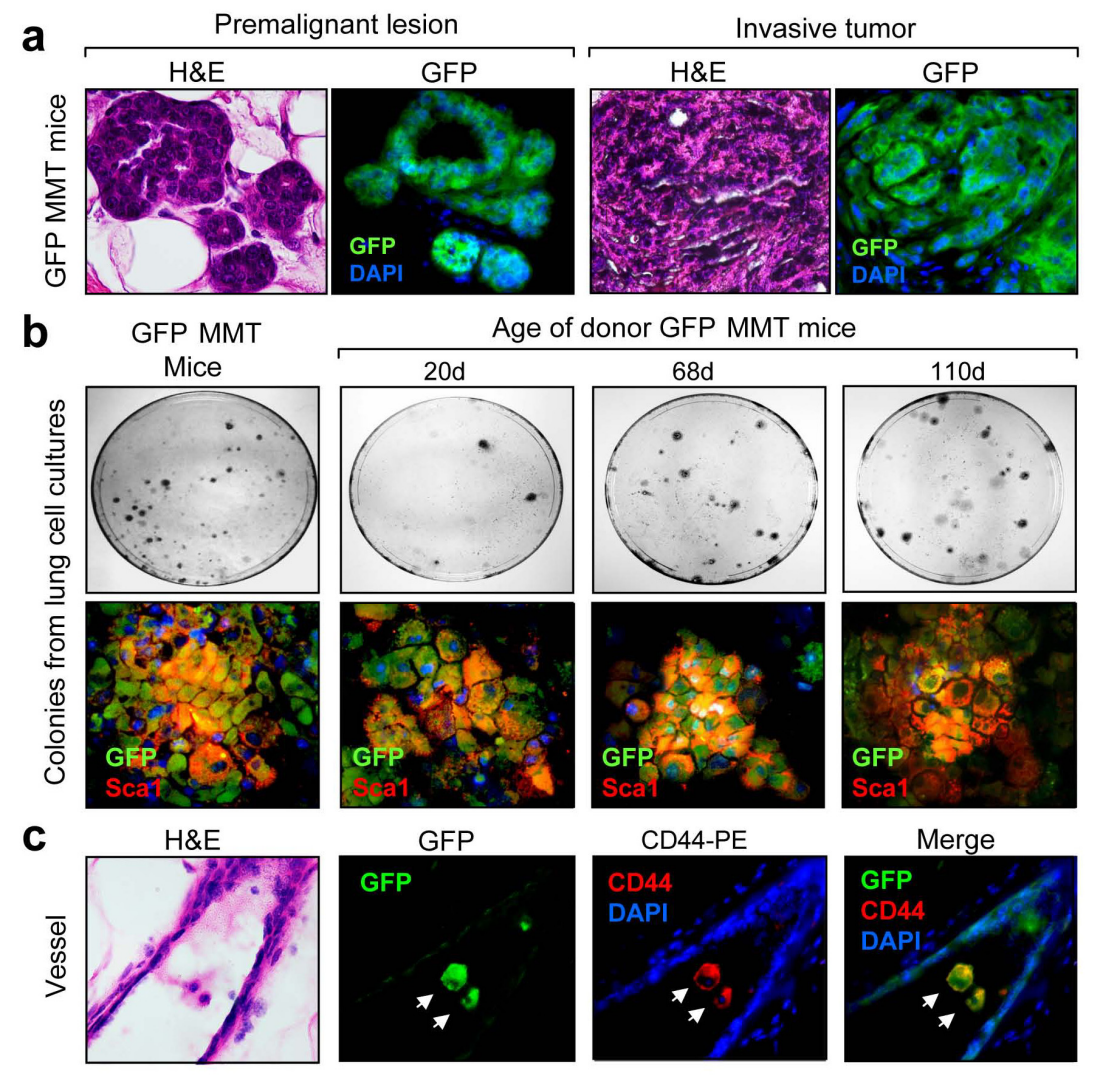
**Detection of disseminated cells in WT recipient mice after inoculation with lung tissue cells from GFP MMT mice**. (**a**) Mammary glands harvested from mice transgenic for PyMT and MUC1 antigen (MMT) that also express green fluorescent protein (GFP MMT) were processed for histological examination under light or fluorescence microscopy (60 x). (**b**) Colony-forming assay. WT mice were injected with lung tissue cells isolated from GFP MMT mice at the indicated ages. Lungs were then collected and perfused to remove circulating cells. Single-cell suspensions from the lungs were cultured in DMEM for two weeks. Colonies with GFP and Sca1 expression were observed under fluorescence microscopy (lower panels). (**c**) Detection of GFP and CD44-positive cells in blood vessels of lung from a WT recipient mouse. Samples from the recipient lungs were also processed for staining with H&E or anti-CD44 monoclonal antibodies.

Our findings of lung metastatic cells in recipient WT mice suggest that disseminated tumor cells should be detectable in various tissues outside the primary tumor, including blood and LNs. To facilitate the identification of the disseminated tumor cells, we next generated green fluorescent protein (GFP) MMT mice. Firstly, in Figure 5a, we showed that the intracellular expression of GFP did not alter mammary tumorigenesis, making this a viable tumor model. However, mammary epithelial/tumor cells expressing GFP were easily identified by fluorescence microscopy in WT mice transplanted with tumor cells from GFP MMT mice. Single cell suspensions of lungs were obtained from GFP MMT mice at various stages of tumorigenesis (after the lungs were perfused to remove the circulating tumor cells), and then transplanted into mammary fat pads of WT mice. Lung cells from old GFP MMT mice were used as a positive control. Sixty days after cell inoculation, the lungs harvested from WT recipient mice were processed for colony-forming assay. As shown in Figure 5b (upper panels), GFP expressing colonies were observed in the lungs of GFP MMT mice as well as WT mice inoculated with lung tissue cells. These colony cells also expressed Sca1 (Figure 5b, lower panels). Interestingly, colonies positive for GFP were observed in cultures of lung tissue cells from mice as young as 20 days old, suggesting that dissemination is an early event in this model of mammary tumorigenesis. Furthermore, GFP expressing cells without or with cell surface CD44 were found inside blood vessels (Figure 5c). These results provide further support that a subpopulation of mammary epithelial cells expressing CD44/Sca1 is endowed with disseminating and metastatic potential.

### Differential tumorigenic and metastatic potential in the subpopulations of lung and mammary epithelial/tumor cells

**Figure 6:**
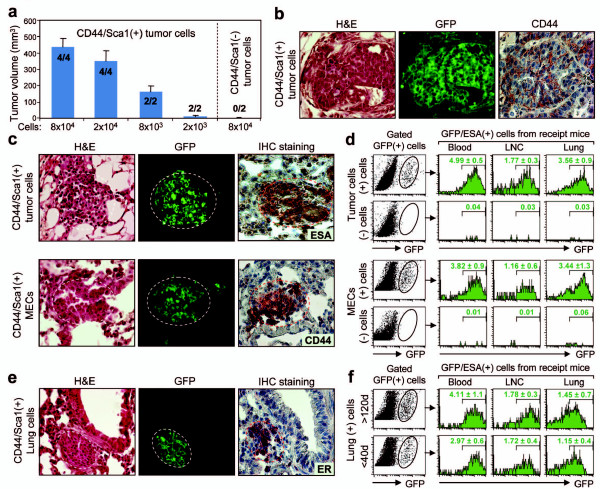
**Tumorigenic and metastatic potential of CD44/Sca1^+ ^cells**. CD44/Sca1^+ ^and CD44/Sca1^- ^cells were obtained by cell sorting from mammary epithelial cells (MECs), tumor cells or the lungs of mice transgenic for PyMT and MUC1 antigen (MMT) that also express green fluorescent protein (GFP MMT) at different stages of tumor development and injected into the mammary fat pads of wild type (WT) mice at the doses indicated (if not indicated in this way, 8 × 10^4 ^cells were injected). Tissue samples were obtained from WT mice injected with either sorted CD44/Sca1^+ ^cells [(+)] or CD44/Sca1^- ^cells [(-)]. (**a**) Comparison of tumorigenic potential between CD44/Sca1^+ ^and CD44/Sca1^- ^tumor cells. The tumor incidence and tumor size are presented for each group of mice inoculated with the indicated numbers of cells. (**b**) Mammary tumor from a WT mouse inoculated with CD44/Sca1^+ ^tumor cells was viewed by H&E staining (left panel) and examined for GFP (middle panel) or CD44 (right panel) expression using immunohistochemical (IHC) staining. (**c**) Sections of mammary glands from WT mice inoculated with CD44/Sca1^+ ^tumor cells (> 120 days, upper panels) or MECs (< 40 days, lower panels) were stained with H&E and examined for expression of GFP and ESA or CD44. (**d**) Quantification of disseminated cells. GFP-positive cells were gated from lung, lymph node cells (LNC) or blood cells of recipient mice inoculated with CD44/Sca1^+ ^and CD44/Sca1^- ^cells from tumor cells or MECs of GFP MMT mice, and stained with anti-ESA monoclonal antibodies and analyzed by FACS. (**e**) Tumors from the lungs of WT mice inoculated with CD44/Sca1^+ ^lung cells from GFP MMT mice at more than 120 days were viewed by H&E staining and examined for GFP and estrogen receptor (ER) expression. (**f**) Quantification of disseminated cells. GFP-positive cells were gated from lung, LNC or blood cells of recipient mice inoculated with CD44/Sca1^+ ^and CD44/Sca1^- ^cells from GFP MMT mice at the indicated ages, stained with anti-ESA monoclonal antibodies and analyzed by FACS. (**d **and **f**) The average ± standard deviation of ESA/GFP-positive cells in total blood, LN or lung cells were presented.

We next determined differential tumorigenic and metastatic potential in cell populations derived from lung or mammary epithelial/tumor cells. CD44/Sca1^+ ^and CD44/Sca1^- ^cell populations were sorted from the mammary glands and lungs of GFP MMT mice at young (< 40 days) or old ages (> 120 days) and then injected into the mammary fat pads of WT mice. Injection of CD44/Sca1^+ ^cells from mammary tumors or lungs of MMT mice resulted in the formation of tumors, regardless of the donor age (Table 1). In contrast, none of the mice inoculated with CD44/Sca1^- ^cells developed tumor. We also performed a cell number titration for tumorigenesis. Injection of as few as 2,000 CD44/Sca1^+ ^cells resulted in the formation of tumors in the recipient mice, while CD44/Sca1^- ^cells failed to form tumors (Figure 6a). Histological examination showed that these tumors were positive for GFP and that some tumor cells were also positive for CD44 (Figure 6b). These results indicate the differential tumorigenic capacity between CD44/Sca1^+ ^and CD44/Sca1^- ^cells.

**Table 1:** Tumorigenic potential of CD44/Sca1-positive cells

Sorted donor cells	Donor age (days)	Numbers of recipient mice	Tumor incidence
Tumor (+) cells	> 120	4	4/4
Tumor (-) cells	> 120	2	0/2
MECs (+) cells	< 40	3	3/3
MECs (-) cells	< 40	2	0/2
Lung (+) cells	> 120	5	5/5
Lung (+) cells	< 40	3	3/3
Lung (-) cells	> 120	2	0/2

CD44/Sca1 positive (+) and CD44/Sca1 negative (-) cells were obtained by cell sorting from tumor cells, mammary epithelial cells (MECs), or lungs of GFP MMT mice at indicated ages and 8 × 10^4 ^cells were injected into the mammary fat pads of wild type mice. Tumor incidence was determined 60 days after cell inoculation.

We next compared the metastatic potential of CD44/Sca1^+ ^and CD44/Sca1^- ^cells. Metastatic lesions were found in the lungs of mice inoculated into the mammary fat pad with CD44/Sca1^+ ^cells but not observed in mice injected in a similar way with CD44/Sca1^- ^tumor cells, regardless of donor age (Figure 6c). These metastatic lesions expressed GFP and ESA or CD44 (Figure 6c). In addition, we quantified the levels of GFP-positive cells in the blood, LN and lungs of recipient mice. As shown in Figure 6d, injection of CD44/Sca1^+ ^tumor cells resulted in the detection of 4.99 ± 0.5%, 1.77 ± 0.3%, and 3.56 ± 0.9% GFP and ESA double positive (GFP/ESA^+^) cells in the blood, LN and lungs, respectively, of recipient mice. In contrast, only 0.04%, 0.03%, and 0.03% GFP/ESA^+ ^cells were detected in the blood, LN, and lungs of mice injected with CD44/Sca1^- ^cells. Similar trends were observed in mice injected with CD44/Sca1 positive or negative MECs from young GFP MMT mice. GFP/ESA^+ ^cells were detected in the blood, LN and lungs of recipient mice injected with CD44/Sca1^+ ^cells. In contrast, few GFP/ESA^+ ^cells were found in these compartments of mice injected with CD44/Sca1^- ^MECs (Figure 6d). Metastatic lesion positive for GFP and ER was also found in the lungs of mice injected with CD44/Sca1^+ ^cells from the lungs of GFP MMT donor mice. In addition, GFP/ESA^+ ^cells were detected in the blood, LN and lungs of recipient mice, regardless of donor age (Figures 6e and 6f). These results further confirm our findings that the CD44/Sca1^+ ^cell population can play a major role in metastasis in MMT mice and that tumorigenic and metastatic potential may exist in cells early in mammary tumorigenesis.

## Discussion

Breast cancer is initiated as a local disease but can also seed metastases to distant organs; indeed the spread of cancer cells from primary tumor sites to distant organs accounts for the majority of deaths in breast cancer patients [1]. Metastasis was thought to be a relatively late event observed at tertiary stages of tumor progression and the result of sequential acquisition of genetic variations in the cells that populate a neoplasm [3]. This hypothesis is supported by the observation that metastasis is rarely discovered in patients with premalignant lesions or non-invasive cancers. However, the absence of metastasis at precancerous stages may not exclude the possibility of dissemination of precancerous cells, which may result in outgrowth and recurrence many years later. Indeed, Engel and colleagues analyzed a total of 12,423 breast cancer patients with a median follow up of 9.4 years. They estimated that the time from initiation of metastases to its diagnosis was 5.8 years [29]. Based on subsequent analysis of 33,000 breast cancer patients, the authors proposed that all metastases are initiated before the removal of primary tumors and suggested a model of parallel growth of primary and secondary tumors [30]. Animal studies by Husemann and colleagues [16] showed disseminated tumor cells and micrometastases in the bone marrows of mice transplanted with premalignant HER-2 transgenic glands. In addition, the authors demonstrated the invasiveness of cells with atypical ductal hyperplasia using electron microscopy, suggesting migration potential for premalignant cells. Using inducible oncogene activation, Posypanina and colleagues [26] showed long-term survival of normal MECs in the lungs. After the oncogenes *myc *and *ras *or *PyMT *became activated by feeding with doxycycline, mammary tumors developed in the lungs within three to four weeks whereas transplantation of retrieved MECs without oncogene activation into mammary fat pad resulted in the formation of mammary ductal trees. Although the MECs were directly injected into the tail vein, thus bypassing the initial steps of metastasis cascade, the authors provide unambiguous evidence at least in their model that relatively normal looking mammary cells can survive and undergo tumorigenesis in ectopic site. These results support the model of parallel development of primary and secondary tumors at least in some malignancies. Consistent with these findings, we have detected disseminated mammary epithelial cells and metastasis in the lungs of MMT mice as early as 26 and 60 days postnatal, respectively (Figure 1). In 60-day-old MMT mice, mammary epithelial cells appear to be at a premalignant stage of progression. These experiments suggest that some MECs from MMT mice are endowed early in tumorigenesis with the capacity to complete the entire metastasic cascade and that they migrate early in tumor development [31]. When lung cells from these mice were injected into the mammary fat pads of WT mice, mammary tumors resembling the autochthonous tumor developed (Figure 3). In addition, tumor cell colonies were obtained from culture of lung tissue cells of recipient mice, suggesting that these cells maintain their metastatic capability in transit from primary MMT mammary tumor to MMT lung metastasis, formation of *de novo *tumors in WT hosts and in founding colonies at distant sites in the recipient WT mouse (Figure 4). The colony-forming assay is a very sensitive method to detect disseminated tumor cells and micrometastasis because only cells with extensive proliferative capacity can form cell colonies [32]. However, normal stem cells also possess unlimited proliferative ability and thus can form cell colonies although we did not observe colony formation in cultures of lung tissue cells from control MUC1.Tg mice (Figure 1c). To allay this concern, we transplanted the lung tissue cells from MMT mice at various stages of tumorigenesis into the mammary fat pads of WT mice. The formation of mammary tumors from these transplants indicates that the colony-forming cells are tumor cells.

We performed three sets of experiments to identify cells responsible for metastasis from primary tumors in MMT mice. We first isolated the lung cells from MMT mice at various stages of tumorigenesis and their tumor initiation and metastasis were confirmed through transplantation in WT mice (Figures 3 and 4). We next phenotyped the lung cells of MMT mice and found that CD44/Sca1^+ ^cells were likely candidates for disseminated or metastatic cells (Figures 2 to 4). We finally identified and sorted the CD44/Sca1^+ ^cells from mammary epithelial/tumor cells of MMT mice at premalignant and malignant stages of tumorigenesis. After transplantation in WT mice, these cells developed primary and metastatic mammary tumors that resembled the parent tumor and primary tumors derived from transplantation with lung cells (Figures 4 and 6 and Table 1), thus linking metastasis from PyMT-induced primary breast carcinoma to a subset of cells with tumor-initiation ability. It could be argued that tumor formation by the lung cells in MMT mice is the result of direct transformation by PyMT rather than disseminated mammary tumor cells. However, the expression of PyMT is under the transcriptional control of the MMTV promoter that is active specifically in mammary epithelial cells. In MMTV-PyMT mice, high levels of expression of the PyMT product were detected in female mammary tumors, with lower levels detected in the ovaries and salivary glands [24]. Although PyMT was detected in the lungs of older PyMT mice, lung-specific expression was not observed in younger mice and is correlated with the appearance of lung metastasis. In addition, the lung tumors observed in our studies resembled mammary tumors [23] (Figure 6). Thus it is unlikely that lung tumors in MMT mice or tumors derived from transplantation of lung cells resulted from the lung epithelial cells induced by PyMT.

The GFP MMT mice may provide a useful model for the study of metastasis of mammary carcinomas. For example, disseminated and metastatic cells at various stages of tumor development can be identified in the various tissue compartments and characterized at the cellular, molecular, and genetic levels. Moreover, the model is particularly useful to evaluate early intervention in the treatment of disseminated tumor cells or micrometastasis. Such studies are currently underway.

Our results are also consistent with predictions under the cancer stem cell hypothesis. We have demonstrated that the MMTV-PyMT murine model of breast cancer yields a consistent progression of spontaneous mammary carcinomas with metastasis to the lung. We have shown that tumorigenesis in the mammary glands of MMT mice parallels the development of metastasis and that cells which express putative stem cell markers may represent a population of cells important for the growth of lung metastasis. We have also found that the subpopulation of transformed mammary cells in the primary site or in lungs to which metastasis has occurred can itself lead to tumor growth in PyMT-negative hosts. These findings suggest that cells with a stem cell phenotype play a causal role in both tumorigenesis and metastasis.

## Conclusions

The tumorigenic and metastatic potential of a subpopulation of spontaneous tumors in MMT mice is endowed relatively early in mammary tumorigenesis and suggests a potential role for cancer stem/progenitor cell sub-populations in metastasis.

## Abbreviations

ANOVA: analysis of variance; CSC: cancer stem cells; DCIS: ductal carcinoma in situ; DMEM: Dulbecco's modification of Eagle's medium; ER: estrogen receptor; ESA: epithelial specific antigen; FCS: fetal calf serum; GFP: green fluorescent protein; H&E: hematoxylin and eosin; ICC: immunocytochemical staining; LN: lymph node; MECs: mammary epithelial cells; MMT: mouse transgenic for PyMT and MUC1; MMTV-LTR: mouse mammary tumor virus long terminal repeat; MUC1: mucin 1; PBS: phosphate-buffered saline; PCR: polymerase chain reaction; pCSC: precancerous stem cells; PyMT: polyomavirus middle-T oncogene; WT: wild type.

## Competing interests

The authors declare that they have no competing interests.

## Authors' contributions

DW participated in the design of the study, carried out experiments including colony forming assay, immunocytochemical staining, assessment of tumorigenic and metastatic capacity of lung metastatic cells in the new host, and performed the statistical analysis. JHP carried out experiments including histological and immunohistochemical staining, colony-forming assay, assessment of tumor formation and metastasis in mice and participated in statistical analysis. BS participated in the design of the study and helped drafting the manuscript. SK contributed to the design of the study. SKC participated in drafting of the manuscript. JG conceived of the study, designed, coordinated and participated in the experiments, and drafted the manuscript. All authors read and approved the manuscript.

## Supplementary Material

Additional file 1 **Before and after lung perfusion**. Lungs were harvested prior to (upper panels) or after perfusion (lower panels). The lung became pale after perfusion and histological examination shows depletion of blood cells. Click here for file
