# Supplementary figures and images for: Metastasis is an early event in mouse mammary carcinomas and is associated with cells bearing stem cell markers
Source: Breast Cancer Res. 2012 Jan 25;14(1):R18. doi: 10.1186/bcr3102 (PMC3496135; doi:10.1186/bcr3102)

Before perfusion

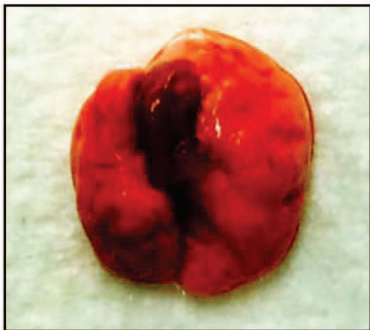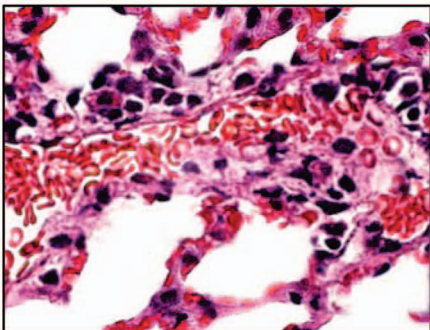

After perfusion

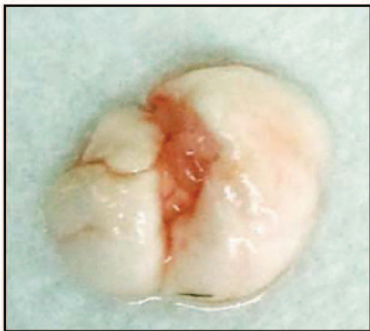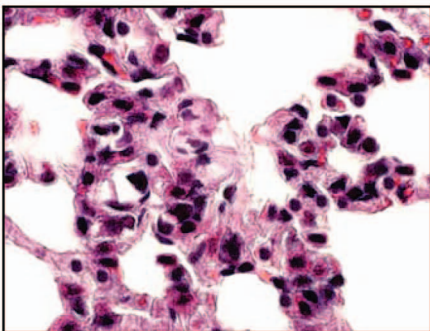

Supplement: Additional file 1 — Before and after lung perfusion. Lungs were harvested prior to (upper panels) or after perfusion (lower panels). The lung became pale after perfusion and histological examination shows depletion of blood cells. [file bcr3102-S1.PDF]
